# Reproductive, maternal, newborn and child health service delivery during conflict in Yemen: a case study

**DOI:** 10.1186/s13031-020-00269-x

**Published:** 2020-05-27

**Authors:** Hannah Tappis, Sarah Elaraby, Shatha Elnakib, Nagiba A. Abdulghani AlShawafi, Huda BaSaleem, Iman Ahmed Saleh Al-Gawfi, Fouad Othman, Fouzia Shafique, Eman Al-Kubati, Nuzhat Rafique, Paul Spiegel

**Affiliations:** 1 Center for Humanitarian Health, Johns Hopkins Center for Humanitarian Health, Baltimore, MD USA; 2 Sana’a University, Sana’a, Yemen; 3 University of Aden, Aden, Yemen; 4 Taiz University, Taiz, Yemen; 5 UNICEF Yemen Country Office, Sana’a, Yemen

**Keywords:** Yemen, Humanitarian, Conflict, War, Health system, Health services, Maternal, Newborn, Child, Reproductive health

## Abstract

**Background:**

Armed conflict, food insecurity, epidemic cholera, economic decline and deterioration of essential public services present overwhelming challenges to population health and well-being in Yemen. Although the majority of the population is in need of humanitarian assistance and civil servants in many areas have not received salaries since 2016, many healthcare providers continue to work, and families continue to need and seek care.

**Methods:**

This case study examines how reproductive, maternal, newborn, child and adolescent health and nutrition (RMNCAH+N) services have been delivered since 2015, and identifies factors influencing implementation of these services in three governorates of Yemen. Content analysis methods were used to analyze publicly available documents and datasets published since 2000 as well as 94 semi-structured individual and group interviews conducted with government officials, humanitarian agency staff and facility-based healthcare providers and six focus group discussions conducted with community health midwives and volunteers in September–October 2018.

**Results:**

Humanitarian response efforts focus on maintaining basic services at functioning facilities, and deploying mobile clinics, outreach teams and community health volunteer networks to address urgent needs where access is possible. Attention to specific aspects of RMNCAH+N varies slightly by location, with differences driven by priorities of government authorities, levels of violence, humanitarian access and availability of qualified human resources. Health services for women and children are generally considered to be a priority; however, cholera control and treatment of acute malnutrition are given precedence over other services along the continuum of care. Although health workers display notable resilience working in difficult conditions, challenges resulting from insecurity, limited functionality of health facilities, and challenges in importation and distribution of supplies limit the availability and quality of services.

**Conclusions:**

Challenges to providing quality RMNCAH+N services in Yemen are formidable, given the nature and scale of humanitarian needs, lack of access due to insecurity, politicization of aid, weak health system capacity, costs of care seeking, and an ongoing cholera epidemic. Greater attention to availability, quality and coordination of RMNCAH services, coupled with investments in health workforce development and supply management are needed to maintain access to life-saving services and mitigate longer term impacts on maternal and child health and development. Lessons learned from Yemen on how to address ongoing primary health care needs during massive epidemics in conflict settings, particularly for women and children, will be important to support other countries faced with similar crises in the future.

## Background

Yemen is one of two countries currently identified by the United Nations (UN) Inter-Agency Standing Committee as a Level 3 Emergency, indicating the need for large-scale system-wide response to meet immediate humanitarian needs [1]. Prior to the Houthi siege of Yemen’s capital in 2014 and escalation of both intra-state and inter-state conflict in March 2015, Yemen was already the poorest country on the Arabian Peninsula, and imported nearly 90% of its medical supplies and fuel [2]. The health system was characterized by significant disparities in coverage, coupled with very limited financial protection, and inequitable distribution of resources [3, 4] As in many other low-income countries, public sector health care worker pay and retention were significant problems; rural areas struggled with limited health infrastructure and workforce shortages while dual job-holding in public and private sector clinics was common in urban areas [5]. Nevertheless, health sector development initiatives resulted in moderate improvements in health service coverage and outcomes between 1997 and 2013 [6].

**Fig. 1:**
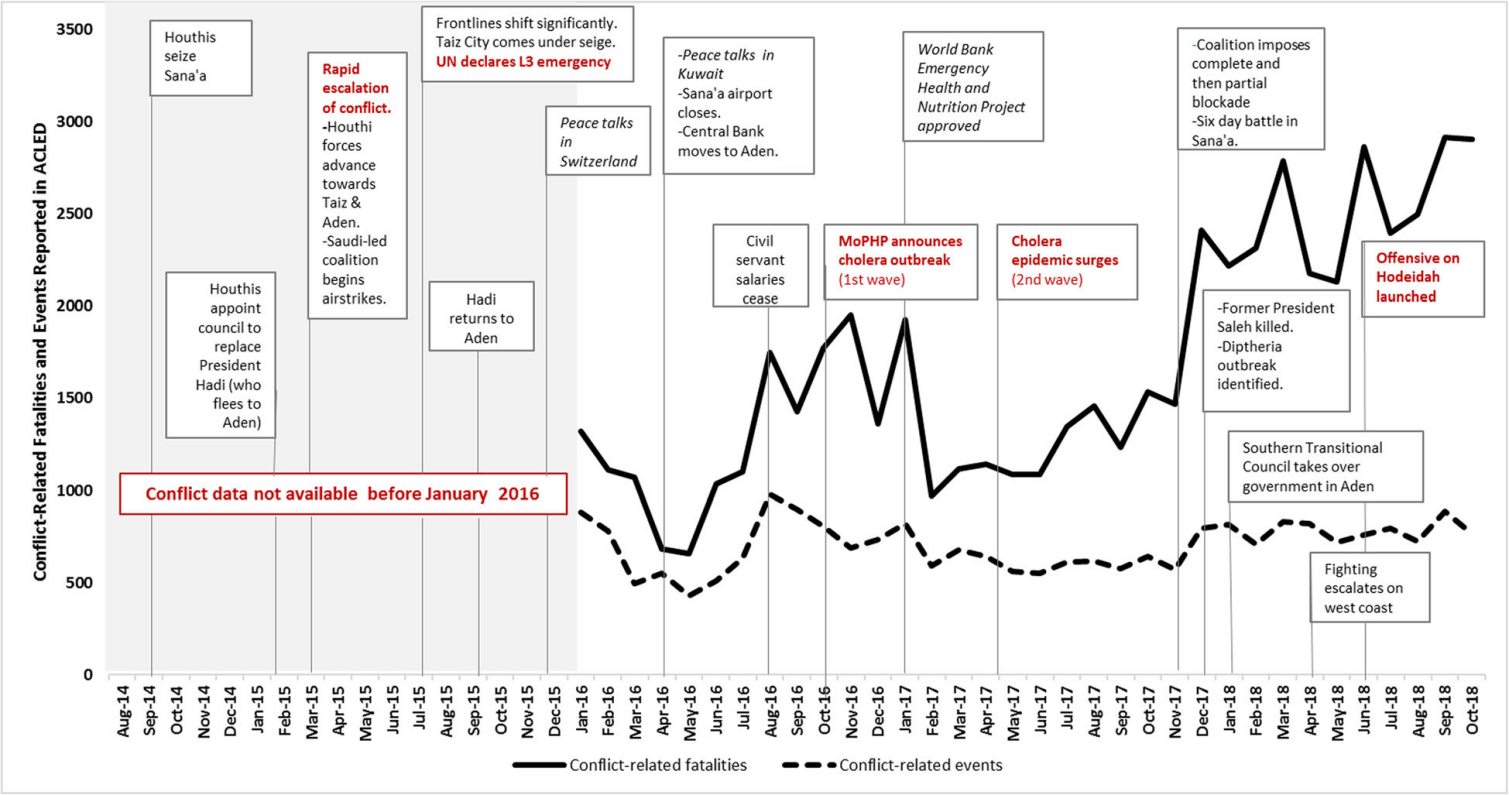
Timeline of key events and intensity of violence

The current conflict, now in its fourth year, has resulted in a complex humanitarian emergency that includes seemingly intractable power struggles, widespread conflict-induced displacement, a slow onset crisis in food security and malnutrition, and one of the largest cholera outbreaks in recent history – among other health and protection challenges [7–9]. Although the international community has responded with approximately six billion dollars of humanitarian aid since 2015, needs have expanded and deepened to the point where, in 2018, nearly 80% of the population of Yemen is in need of humanitarian assistance, 10% of the population is internally displaced, and the country is at the brink of a famine [10–12]. Violence and insecurity have ebbed and flowed over time (Fig. 1), with parties to the conflict drawing international condemnation for repeated attacks against civilians and civilian infrastructure - including health facilities, water and sanitation infrastructure, schools and roads and bridges [9]. The Armed Conflict Location and Event Database (ACLED) reports more than 57,000 conflict-related fatalities (including civilians and non-civilians) between January 2016 and October 2018 [13], and human rights groups have documented more than 200 attacks on health infrastructure, including airstrikes, shelling, occupation and looting of health facilities, hijacking ambulances, and threats or attacks on health workers [9, 14]

Reports over the last four years have described the health system as “devastated”, “on the brink of collapse”, and “failed under relentless pressure” of conflict and multiple disease outbreaks [8, 15–20]. In early 2016, the World Health Organization assessed 3507 health facilities in 16 of Yemen’s 22 governorates, finding only 45% fully functional, 38% partially functional and 17% not functional at all [21]. Approximately 1.25 million civil servants, including health workers, have not received salary since August 2016 [22]. Blockades of entry ports and mismanagement of humanitarian aid within Yemen has exacerbated food insecurity and severely limited the availability of resources needed to maintain routine primary health care services or respond to outbreaks [19, 23, 24]. As general livelihoods have deteriorated, so has the population’s ability to afford health care. Recent reports indicate excess mortality among children, mothers and patients suffering from communicable diseases, malnutrition, non-communicable diseases, or those who cannot access care because of the conflict [10]. For example, recent estimates suggest than nearly 85,000 children under age 5 may have died from extreme hunger or disease since the escalation of conflict in early 2015; children under age 15 also accounted for 41% of suspected cholera cases and approximately 1 in 4 deaths [25, 26].

Although numerous advocacy reports and commentaries have been published, health research conducted over the last four years in Yemen is limited. The most recent national survey of health service coverage was the 2013 Yemen Demographic and Health Survey [6]. Food security and nutrition surveys were conducted in 2014 and 2016, but do not provide information on broader health status or service coverage indicators [27, 28]. The few studies published include rigorous analysis of cholera surveillance data, [29, 30] a review of health system functionality prior to the escalation of conflict [3], an evaluation of a family planning and post-abortion care program in two governorates, [31, 32] and modeling of potential impacts of conflict on health service delivery [33].

This case study examines how reproductive, maternal, newborn, child and adolescent health and nutrition (RMNCAH+N) services have been delivered and identifies factors influencing implementation in three governorates of Yemen. It is one of 10 case studies conducted by the BRANCH (Bridging Research and Action in Conflict Settings for the Health of Women and Children) Consortium to examine RMNCAH+N service coverage and implementation strategies in conflict-affected settings [34].

## Methods

We used a case study design, modified from a standardized protocol agreed upon by the BRANCH consortium and adapted for implementation in Yemen. Multiple data sources, including peer-reviewed and grey literature and datasets identified through a desk review as well as qualitative data from key informant interviews and focus group discussions, were analyzed to gain insights into how programmatic and contextual factors have influenced provision of RMNCAH+N services since the escalation of conflict in 2015.

### Study setting

This case study focuses on three of the country’s 22 governorates, selected in consultation with the Ministry of Public Health and Population and UNICEF.

- Sana’a City (population 3 million) is the capital of Yemen recognized in the Constitution, and its largest city. Sana’a City has been under Houthi-led government control since September 2014.
- Aden (population 925,000) was the capital of South Yemen (1967–1990) and is the fourth largest city in Yemen. Parts of the city were under Houthi control from March – July 2015. Since then, the city has been under the control of the internationally-recognized government, supported by a Saudi-led coalition of international actors. It is the main cargo seaport for Yemen.
- Taiz (population 3.2 million) is Yemen’s most populous governorate, home to Yemen’s third largest city (Taiz city), as well as rural highland and lowland areas. Taiz has been a frontline of the conflict since March 2015, with multiple changes in control of territory within the governorate. At the time of data collection, 13 districts were controlled by the internationally recognized government, 6 districts by the Houthi-led government, and 4 districts actively contested.

These governorates were selected to reflect variation in population and health system characteristics, as well as the nature of conflict. Sana’a City and Aden, both densely populated urban governorates with notably higher reproductive, maternal and child health services coverage, were selected to ensure areas under control of the Houthi-led government and internationally-recognized government were represented, while Taiz, with a more dispersed and underserved population, was selected due to the intensity of recent and ongoing conflict, population size, level of humanitarian presence, and accessibility for data collection. Nearly 1 in 4 conflict-related fatalities (23%) reported since January 2016 occurred in Taiz; while only 1.5% of fatalities occurred in Aden and 1.7% of fatalities occurred in Sana’a City during the same period [13].

**Fig. 2:**
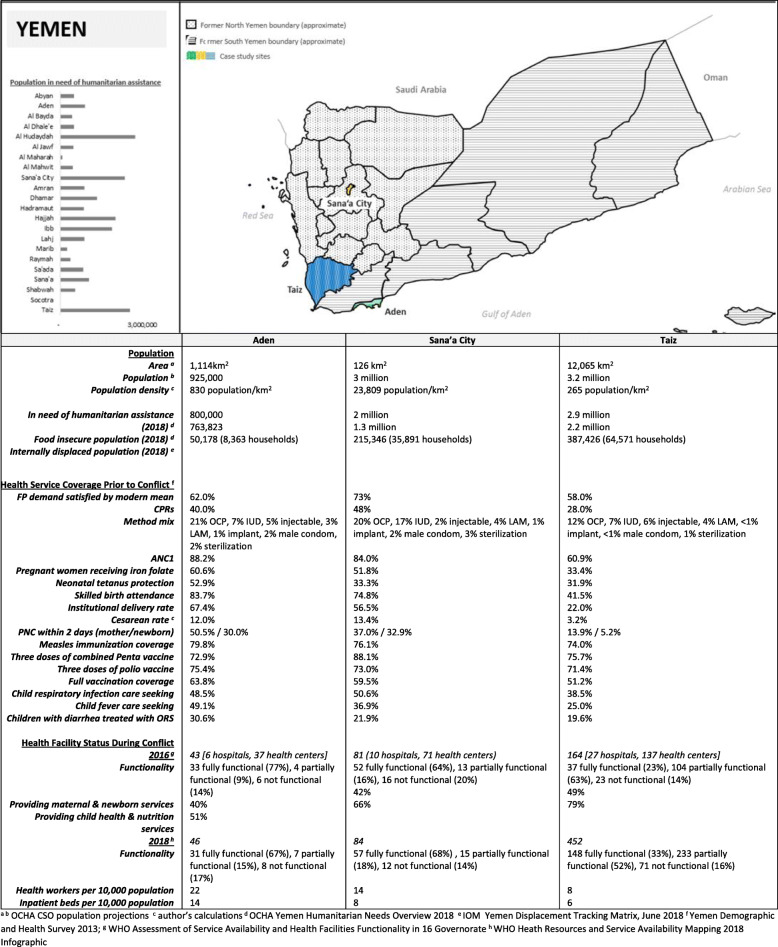
Demographic and health characteristics of selected study sites

**Fig. 3:**
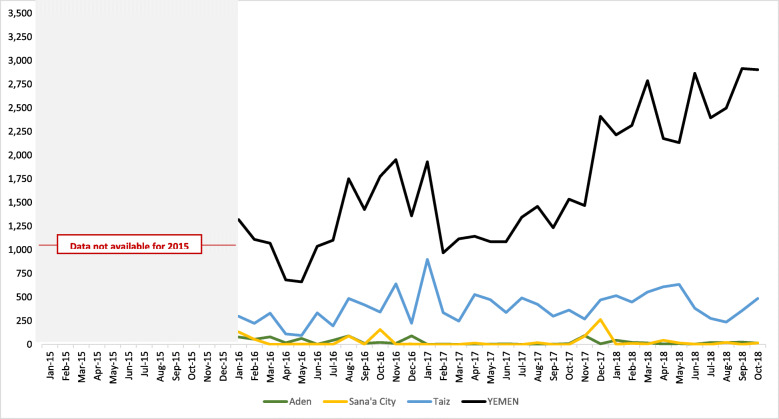
Conflict-related fatalities in case study governorates, January 2015 – October 2018 [13]

Demographic and health characteristics of each governorate are summarized in Fig. 2. and the intensity of conflict over time, is shown in Fig. 3.

### Data collection and analysis

#### Literature and data review

A systematic search of peer-reviewed and grey literature published since 2000 was conducted to compile information on RMNCAH+N services in Yemen before/after the escalation of conflict in 2015 and identify datasets with information on RMNCAH+N status and service coverage in each of the study sites. (Supplemental file 1) All documents and data sources received were cataloged and screened for relevance to case study research questions. Included documents were then reviewed by study team members; relevant text content was coded using in NVivo 12 and quantitative indicators of interest were summarized using a standardized data extraction form [35]. Quantitative analysis focused on examination of data quality and consistency, and descriptive comparison. No further quantitative analysis was conducted.

#### Primary qualitative data collection and analysis

Primary qualitative data collection included 94 semi-structured individual and group in-depth interviews (IDIs) and six focus group discussions (FGDs) conducted in September–October 2018. A total of 181 individuals participated in the study, including 27 national and sub-national government authorities, 50 humanitarian agency staff, and 104 healthcare providers across the three governorates. (Table 1). All interviews with government authorities and healthcare providers were individual interviews. Interviews with humanitarian agency staff were conducted with at least one, and as many as 5 people per interview to ensure the breadth of agency engagement in RMNCAH+N services was represented. Within each governorate, data collection sites (e.g. districts, health facilities and communities) were purposively sampled to reflect diversity. At each site, convenience sampling methods were used to select both senior and mid-level providers that could offer a diverse range of perspectives on the RMNCAH+N continuum of care. Facility-based healthcare providers included two specifically responsible for family planning and other reproductive health services, 16 primarily responsible for maternal and newborn health service provision, three immunization specialists, five child healthcare providers, five nutrition specialists and 12 healthcare managers or generalists. Similarly, humanitarian agency staff and government officials included both technical specialists and broader health and nutrition program managers and policy makers. In Taiz, healthcare providers in both internationally-recognized and de-facto government controlled areas were included. Demographic characteristics of participants were not recorded, to protect anonymity of participants. Data collection tools (Supplementary File 2) were developed based on common data collection guidelines for the ten case studies and adapted for the health system and humanitarian crisis in Yemen. All tools were translated to and back-translated from Arabic before training of data collectors. All data collectors held at least a bachelor’s degree and were local to their governorates. All received five days of training covering the study objectives, data collection procedures and interview guides as well as qualitative interviewing skills and research ethics. Data collection was done in Yemeni-dialect Arabic. All interviews and FGDs were audio recorded after consent of participants except for four IDIs, where the participant refused. Audio recordings were transferred to US-based study team members and transcribed in English by a US-based consultant firm.

**Table 1:** Qualitative data collection activities and participants by governorate

	Aden	Sana’a City	Taiz	TOTAL
**Government authorities**	7 IDIs − 1 MoPHP official − 6 Governorate health office officials	11 IDIs −5 MoPHP officials − 6 Governorate health office officials	9 IDIs − 7 officials in internationally-recognized government-controlled areas − 2 officials in Houthi-controlled or contested areas	**27 IDIs**
**UN and NGO health program managers**	8 IDIs (21 participants) −3 UN agencies − 2 INGOs − 3 national NGOs	11 IDIs (22 participants) − 3 UN agencies − 2 INGOs − 2 national NGOs	5 IDIs (7 participants) − 1 UN agency − 2 INGOs − 1 national NGO	**24 IDIs** (50 participants) − 3 UN agencies − 7 INGOs − 6 national NGOs
**Facility-based healthcare providers** **(**1 management + at least 2 midlevel providers per facility)	13 IDIs [3 facilities] − 6 hospital staff, 7 primary healthcare facility staff	11 IDIs [3 facilities] − 5 hospital staff, 6 primary healthcare facility staff	19 IDIs [5 facilities – including 3 in internationally-recognized government-controlled areas and 2 in Houthi-controlled areas] − 13 hospital staff, 6 primary healthcare facility staff	**43 IDIs** [11 facilities]
**Community-based healthcare providers**	2 FGDs (20 participants) − 10 community midwives − 10 community health volunteers	2 FGDs (19 participants) − 10 community midwives − 9 community health volunteers	2 FGDs (22 participants) − 10 in internationally-recognized government-controlled area − 12 in Houthi-controlled area	**6 FGDs** (61 individuals trained as community midwives or community health volunteers)
**Total**	**61 individuals**	**63 individuals**	**57 individuals**	**181 individuals**

Content analysis methods were used to analyze transcripts. An analytical codebook was initially developed with deductive codes based on the study objectives and validated by the study team. This was expanded with inductive codes based on Arabic expanded field notes, frequent debriefings with field team members and initial coding of transcripts. All coding of English translated transcripts was done by the second and third authors, who have graduate-level training in public health and are fluent in Arabic, using Nvivo 12 [35]. This phase involved continuous revisions and refinement of the codebook, check-ins for consistency and reflective memos. During the analysis process, the study team discussed codes and themes arising from the data, drawing comparisons across participants’ roles, type of health services and governorates to triangulate findings, and to understand texture and nuance of participant perspectives.

### Synthesis of findings

Desk review and primary qualitative research findings were analyzed separately, then synthesized for a more comprehensive understanding of factors affecting RMNCAH+N service delivery. Desk review findings were primarily used to contextualize and aid in interpretation of qualitative results. Preliminary findings were reviewed with in-country co-investigators from each governorate to clarify, revise and ensure credibility and dependability of the findings to their firsthand experiences.

### Ethical considerations

The Johns Hopkins Bloomberg School of Public Health determined that this study was not human subjects research and therefore did not require institutional review board oversight (IRB 8665). In Yemen, the study protocol was reviewed and approved by the Research and Ethics Committee at the University of Aden School of Medicine and Health Sciences (REC-33-2018). Oral consent was obtained from all study participants before initiating data collection.

## Results

The near collapse of the national health system, coupled with varied levels of humanitarian access challenges across study sites, limits availability of consistent, representative data to assess changes in health service availability, coverage and quality since the escalation of conflict in 2015. Humanitarian agency staff and healthcare providers interviewed in the three study sites provided numerous examples of how the combination of insecurity, reduced availability of health services, and impacts of the economic downturn, has affected both service delivery and care-seeking patterns. Desk review findings reinforce the complexity of challenges in maintaining available health services, and persistent gaps in addressing population health needs. For example, while WHO’s Health Resource Availability Mapping conducted in 2018 shows improvements in the percentage of facilities that are fully functional (from 45% in 2016 to 50% in 2018) and proportion of hospitals providing reproductive health services (from 41% in 2016 to 54% in 2018), the percentage of health centers providing reproductive health services dropped from 40 to 26% in the same period.

While general factors affecting RMNCAH+N service delivery were fairly similar across study sites, the impacts of these factors were strongly influenced by pre-existing health system capacity as well as the nature and intensity of conflict in each governorate. In the sections that follow, common themes are presented, with differences in severity or prominence across study sites noted where applicable.

### Planning and coordination of health services

#### Health sector leadership and governance - a tale of two governments

Both the internationally recognized government, based in Aden, and the de-facto government, based in Sana’a, have MoPHPs that consider themselves responsible authorities for planning and coordination of health services. In Sana’a City, some officials with responsibility for RMNCAH+N services described program planning as short-term and reactionary – based on the types of funds provided for humanitarian assistance and, in some cases, on the political agendas or subjective priorities of “decision-makers” (referring to senior MoPHP leadership or other departments of the de-facto government). As one health official explained, *“sometimes there are some wrong political decisions taken by the leaders of the country. Vaccination campaigns are sometimes prohibited. Leaders tell us that these campaigns are not important and funds can be used for other purposes of higher priority to the country”.* In Aden, participants painted a picture of a profoundly weak MoPHP, with limited capacity to oversee and support health directorates. Communications with directorates are inconsistent, and requests made by the directorates often go unanswered.

Across all three governorates, study participants reported that MoPHP ownership over health program plans was further limited by confusion caused by changes and, at times, duplication in reporting obligations to Sana’a and Aden-based authorities. Deterioration of authority was evident in all three governorates but appeared to be most pronounced in Taiz. The multiplicity of the actors involved – rebel groups, Houthi militants, the Yemeni military – exacerbated governance constraints, with notable impacts on humanitarian assistance and health service delivery. As an MoPHP official in Taiz lamented, *“The [internationally recognized] government appoints a manager and the Ministry in Sana’a appoints another one. So here you find two managers for the same health office”.*

#### Humanitarian response coordination

Documents reviewed indicate that the humanitarian cluster system was activated to coordinate humanitarian activities in Yemen in 2009, with the Nutrition Cluster (co-led by UNICEF and MoPHP) in place from the outset and Health Cluster (co-led by WHO and an international NGO) activated in 2011. Since the escalation of conflict and declaration of an L3 emergency in 2015, sectoral clusters have operated at a national and sub-national level, with activities coordinated through regional hubs in Sana’a City, Aden, Ibb (covering Ibb and Taiz), Sa’ada and Hodeidah. The number of UN, international and national non-governmental organizations participating in Health and Nutrition Clusters has varied from year to year and across governorates. At the end of 2018, there were 8 active Health Cluster member organization in Aden, 10 working in Sana’a City and 17 working in Taiz.

According to documents reviewed and key informants, humanitarian health response efforts, guided by an annual inter-agency Humanitarian Response Plan, focus on maintaining basic health services at functioning facilities, and deploying mobile clinics, outreach teams and community health worker networks (including both community-based midwives and community health volunteers) to address urgent needs in areas with no functional health facility or limited humanitarian access. WHO was the largest non-governmental recipient of funding for implementation of health components of the Humanitarian Response Plan, reporting $172 million in contributions for 2015–2018. UNICEF was the next largest non-governmental aid recipient, reporting a total of $99.9 million in contributions for 2015–2018. UNFPA received a total of $29.2 million for 2015–2018, nearly half of which was received in 2015 [36]. UN staff participating in key informant interviews emphasized that they are a *“provider of last resort”*, lamenting the fact that they are *“replacing”* or *“taking on the role of”* government in allocating resources for health facility running costs.

#### Program planning and decision making

There was general agreement among MoPHP officials in both internationally recognized and de-facto government-controlled areas that they lacked decision-making capacity and that their authority diminished significantly since the escalation of conflict in 2015. Across all study sites, interviews revealed a general frustration by health officials with the receding, or even disappearing, role of their governmental positions in dictating healthcare priorities, funding strategies and staff policies.

*“You have a priority, but the donor has another … [international organizations] focus on indicators. For example, they focus on malnutrition, deworming and vitamin A for children, along with the number of these children and percentage receiving consultations. These things are included in their interest. Any activities, such as training and supervision that benefit these indicators are supported, while infrastructure general management and programs support are not” – MoPHP official, Taiz.*

The link between funding and decision-making was pervasive; officials repeatedly problematized the absence of funds, explaining that their plans could not be implemented due to the public budget deficit and unwillingness of most donors to channel funds through government.

Both MOPHP authorities and NGOs saw UN agencies, or the Health and Nutrition Clusters, as leading humanitarian response efforts, defining strategies for health sector support and resource allocation. Although some UN staff noted the ability to rapidly mobilize funds when needed, other respondents noted earmarking of funds as a barrier to effective humanitarian assistance. Both UN agencies and international NGOs frequently referred to government (beyond MoPHP) as holding ultimate authority on what humanitarian assistance is provided, where, when and how. Across all study sites, maintaining positive relationships with national, governorate and district level authorities is seen as critical for issuance of work permits, selection of staff and program sites, permissions to travel or move supplies within and across governorates.

#### Prioritization of health services for women and children

Health Cluster leadership and representatives of member organizations generally described priorities as *“meeting basic needs”* and *“keeping basic services functioning”*, with some further explaining that this must take precedence over longer term investments in health system strengthening.

With respect to technical priorities, outbreak response and treatment of acute malnutrition were seen as top concerns, deflecting attention away from other routine primary health care services. This dynamic was most apparent in Taiz, where respondents placed greater emphasis on spread of communicable diseases and nutrition needs. In Sana’a City and Aden, urban centers away from the frontlines of the conflict, there seems to be more consistent attention to a broader range of women’s and children’s health needs, and particular focus on emergency obstetric and newborn care. Key informant interview and focus group discussion participants in all three governorates consistently noted that family planning has been neglected; it is not seen as a priority or urgent need in comparison to disease outbreaks. Adolescent health programming was not mentioned by respondents, and when asked about it, most were unaware of any programs or health services specifically for adolescents.

### Service delivery challenges and coping mechanisms

The primary factors affecting RMNCAH+N service delivery and intervention coverage in the three governorates studied appear to be insecurity, politicization of aid, health system capacity, and cost barriers to care seeking. These factors have been persistent since the start of the crisis, but severity and impacts have shifted over time in each governorate. Table 2 summarizes the most common challenges expressed by study participants and current coping mechanisms, where applicable.

**Table 2:** RMNCAH+N service delivery challenges and coping mechanisms

Challenge	Current coping mechanisms	RMNCAH+N services affected^a^
**Scale and urgency of needs**	Prioritization of nutrition and disease control Integrated famine risk reduction strategy Performance-based incentives	All
**Insecurity and humanitarian access constraints**	Use of mobile teams, community midwife and volunteer networks, outreach campaigns to reach specific populations when access permits Reliance on local NGO implementers and third party monitoring Short-term project cycles Coordination of implementation plans	Health education Immunization Sick child care Nutrition screening Family planning
**Availability, retention and motivation of qualified health workers**	Humanitarian agencies contracting staff for specific facilities/projects or providing either individual or facility-level performance-based incentive payments Task shifting In-service and on-the-job trainings	Maternal and newborn care Treatment of acute malnutrition
**Lack of infrastructure and irregularity of supplies**	Humanitarian agencies providing in-kind resources (e.g. fuel) and/or facility-level performance-based incentive payments Payment of a lump sum to HF in charge for meeting water, electricity, security and cleaning needs of the facilities Charging informal user fees and/or requiring patients to purchase supplies Delay or interruption of services	Antenatal care Routine labor and delivery care Emergency obstetric and newborn care Immunization Sick child care Family planning
**Access and affordability**	Selective and delayed care seeking Reliance on community-based care providers	Maternal and newborn care Sick child care Treatment of acute malnutrition
**Distrust and lack of demand**	Advocacy with authorities Awareness campaigns Relocation of services	Immunization Family planning

^a^Challenges affect all RMNCAH + N services: those listed are the areas key informants highlighted as particularly affected

#### Insecurity

Despite special protections afforded to medical facilities and educational, cultural and religious sites under international humanitarian law, many health facilities and warehouses have been damaged or destroyed by air strikes, shelling and looting. Participants described ways in which they have been directly affected by the conflict, ranging from injuries, threats, fear for safety of family members, and destruction of property, to changes in their professional responsibilities and work environments. Examples of direct impact on security on participants across governorates include:

*“Currently, I feel more stress as sometimes we go out while planes are flying overhead in the sky. My house has been affected where a rocket has fallen next to it as the way leads to the military hospital, which is in front of my house.”* -MoPHP official, Sana’a

*“I lost one of my family members … We couldn't help him or go to any medical center because of the clashes, shelling and shooting, and because the area we live in was besieged, he was in need of oxygen. His skin turned blue and he died because we couldn't move him to the hospital.”* -Healthcare provider, Taiz

*“I receive threatening messages, saying they’d hang my head on the gates of the hospital. I received lots of threats. People would advise me to have mercy on myself and my family”*

-Healthcare provider, Aden

Against this backdrop of insecurity, health workers cited constant feelings of apprehension and stress about safety. Hospital managers complained that their facilities lacked security personnel, and that they often had to play that role, checking who enters the hospital and making sure that the patients were safe. There were several reports of health staff being terrorized and intimidated by militants and soldiers who enter health facilities demanding payment for protection, free medication, or priority treatment of their families or kin. Providers spoke to the haphazardness of the attacks, explaining that in most instances they were not aware who the militants were, what they wanted or what their political affiliation was. Key informants from humanitarian agencies also recognized the increased exposure and risks faced by outreach workers, noting attacks on immunization outreach workers, and midwives killed while traveling through insecure areas to reach women in labor or accompany clients to facilities.

During data collection, participants noted that security has improved over time in all three governorates, but challenges persist. In Aden, the most insecure period was between March and July/August of 2015, where Houthi forces controlled portions of the city. In Sana’a, 2015 was the most challenging period. Taiz remains challenged by high levels of insecurity, particularly in contested areas, although comparatively there is a perceived improvement compared to the period between March 2015 and late 2016 as areas of control shifted. A health worker in Taiz city, describing the impact of changes in security situation on care-seeking shared:

*“..now people refuse to come to [Hospital name] because there is a military barracks here in this area, although we do good services, but they refuse to come here because of security concerns … The security situation is very bad here, for example, there was clashes and heavy firing of bullets in the hospital, and everyone was scared here and this spread fear and insecurity … . The situation here has become very tragic and catastrophic. … . most families refuse to go to health centers because of the serious security situation, but when the situation calms down people come and seek health care services. Therefore, the number of patients increases or sometimes decreases according to the security situation. But the security situation has improved recently.”*

In Sana’a and Aden, impacts of insecurity are more indirect. These include unpredictability and effects of airstrikes (in Sana’a), checkpoints, threats by militant groups and an increasingly armed population since the start of the war on staff motivation and client care seeking behaviors.

#### Politicization of aid

Import clearance procedure and challenges moving supplies within Yemen are major challenges. Humanitarian aid flows are often used as a tool to demonstrate geopolitical power, at the expense of population health. For example, participants provided examples of MoPHP authorities blocking immunization campaigns and requesting funds be redirected for other purposes. Similarly, contraceptive commodity distribution has been deliberately blocked by MoPHP authorities in Sana’a; UNFPA reported more than one year of delays in procurement procedures and prolonged negotiations for release of contraceptive commodities from ports. NGOs also consistently highlighted the tight control that national and sub-national government authorities (and in some cases non-state actors) maintain over day-to-day program activities. Examples ranged from permissions to work in certain areas to selection of health facility staff and community volunteers to which health services can be provided.

*“We have recently faced the problem of political interventions in our activities. For example, when we wanted to implement projects in Taiz, the officials said to us ‘why are you implementing these projects in the resistance areas’ and they prevented us from implementing these projects, so we are facing difficulties in implementing programs and projects for political and ethnic reasons … . We are trying to coordinate with all parties in the northern and southern provinces and to satisfy both parties. We had to replace some of the neediest centers where we would carry out our activities with other centers in the northern governorates.”*

-INGO country office

### Health system capacity

Healthcare providers participating in key informant interviews reported surges in demand for services from displaced populations as well as residents increasingly turning to public sector facilities directly managed or supported by international organizations as costs of care in the private sector became untenable. At referral facilities with functioning maternity wards, providers reported insufficient staff, lack of resources, and multiple clients to a bed with poor working conditions and infection prevention. At the same time, humanitarian agency staff and community-based healthcare providers in all three governorates mentioned increases in home births, noting women traditionally prefer to give birth at home and, given access challenges in both urban and peri-urban and rural areas, now tend to seek care only when experiencing complications. Key informants also highlighted the risks this presents, explaining that there is no formal referral system for women needing emergency obstetric care; interview participants in Taiz noted that community midwives know to refer women exhibiting risk factors or danger signs but have no mechanism to ensure women can reach facilities in a safe and timely manner, especially in actively contested areas.

#### Availability, retention and motivation of qualified health workers

High health facility staff turnover was highlighted as a challenge in all three governorates, particularly in urban centers. Primary reasons cited for turnover were economic, including health workers seeking employment outside of Yemen, choosing to work solely at private clinics rather than maintain dual practice (working half days, or 2–3 days per week at public hospitals), higher salaries for programmatic positions at humanitarian agencies, or – for health workers on short-term contracts – shifting to positions with higher or more consistent incentive payments. As one healthcare provider explained:

*“With cholera, the response was big in the first time and many organizations intervened and a lot of money was paid to health staff, so they left other services. For example, a health worker working in nutrition and vaccination left and went to work in an ORC [oral rehydration corner] because he is paid double and the service he is working on decreased. At a certain time, it was affected of course. That improved the second time because ORC decreased. It took a big number of workers and there was more support for workers working in vaccination services.”*

-Healthcare provider, Sana’a

Unequal rates of incentives provided by humanitarian organizations fosters tension among volunteers and negatively impacts their motivation to work. In de-facto government-controlled areas, palpable tensions arose between long-term staff on facility payrolls and short-term contractors. Interviews with government officials revealed their discontent with the role of international organizations in providing incentives, with some claiming that they were creating perverse enticements whereby health workers were only *“working for the money”* or were shifting from one job to another based on the size of the stipend. At the time of data collection, health and nutrition clusters were in the process of developing standardized incentive rates, to mitigate this problem.

Even when staff are present, in many cases they lack the competencies needed to effectively provide services for which they are responsiblela. Thus, rapid orientation and refresher trainings on immunization, integrated management of childhood illness and nutrition services are a core component of many humanitarian response projects, and rapid trainings on aspects of emergency obstetric and newborn care are included in projects that provide support to hospitals with functioning maternity wards. Qualitative findings suggest that health facilities are increasingly relying on midwives and/or recent board graduates to provide care at hospitals, health care centers and in the community, and also that midwives are being called on to provide a broader range of primary health care services, presumably making them less available for maternal and newborn care. Community midwives reported working at public and private clinics, and in their own homes or communities, in a broad range of roles including as vaccinators, nutrition clinic staff, and health educators.

#### Damaged and neglected infrastructure

The destruction of infrastructure was evident across all study sites. Government officials and health workers interviewed commented on damages to health facilities, challenges providing services without consistent electricity, running water or supplies, and failing communication networks as challenges limiting service provision capacity, especially in the case of obstetric emergencies. Facility managers reported an absence of budget allocations for maintenance, repairs and renovations. This spanned basic maintenance such as changing light bulbs and procuring computer devices and printers as well as fixing failing incubators and ultrasound machines.

Respondents also spoke to the impact of long and recurrent electricity cuts on the storage of vaccines and medication. In one example, de-facto government officials recounted how they had to move vaccines from one governorate to another in an effort to keep them safe. In another, a respondent described individual efforts aimed at preserving the vaccine cold chain which ranged from staff bringing in gas cylinders from their homes and installing them to others attempting to fix generators themselves.

Some health care providers and humanitarian agency staff noted that to cope with facility resource limitations, some public health facilities have begun to request fees for services, despite governmental policies of free care. Healthcare providers explained that the income from provision of services is used to pay for diesel for the generators, buy necessary supplies and cover operational costs that are not supported by international donors.

#### Irregularity of supplies

Deficiencies and irregularity of supplies was a common complaint from healthcare providers in all governorates. Extended stock-outs of contraceptives were reported, due to government obstruction of humanitarian imports.

*“[Family planning] is weakly provided due to the lack of supplies. We only provide oral contraceptive pills. Implants haven't been provided since more than two years. IUD and other family planning tools are also weakly provided due to the lack of supplies, and the difficulty of sending them to the directorate and transferring them to health centers.”*

-MoPHP official, Taiz

Some service providers also cited a mismatch between supply of medicines and service demands. In one example, a nutrition department manager of a health facility in Taiz described the lack of medications, adding that the only available medications were antidiarrheal and cholera medicines. These medicines were present in substantial amounts, but they were the only supplies in the entire facility.

The lack of medication has forced clinicians to ask patients to purchase medication from outside of the health facility, which are in turn not always available, may be of questionable or substandard quality or are prohibitively expensive. This has exacerbated the vulnerability of patients who are unable to find or afford medication in private pharmacies. Healthcare workers cited several examples of patients who will come back to the facility after their conditions have worsened and it is clear that they had never bought the prescribed medicines to begin with.

In some instances, shortages in essential medication have created tensions between service providers and members of the community. There were numerous accounts of clashes erupting in medical facilities between patients and service providers. Patients ascribed blame for the shortages in medicines to service providers, accusing them in some cases of stealing the medication.

### Barriers to care seeking

#### Access and affordability

Health seeking behaviors have been severely affected by the crisis. Although we did not interview recipients of care directly, healthcare providers, humanitarian staff and ministry officials shared their insights on factors affecting care-seeking decisions and effectiveness. Several community health volunteers and midwives recounted how they were the only available resource for women and children in their areas, particularly during the height of conflict where most facilities were closed. Healthcare providers and humanitarian agency staff consistently noted that beneficiaries have a hard time accessing facilities due to increased distance required to reach functional facilities, high costs of transportation and services, and in some cases insecurity – including roadblocks and checkpoints.

Financial access seems to be an even greater barrier to care seeking than physical access in many cases. In Sana’a and Aden, health facility managers noted qualified staff leaving the country or seeking employment outside of the health sector as working in private facilities to subsidize work at public hospitals becomes less profitable because clients can no longer afford to pay for care. Across all governorates, the crisis has left many families with no source of income and with the extreme inflation they can no longer afford basic needs. Families cannot afford transportation fees, let alone cost of supplies and medication. This has a direct impact on outcomes of patients who only access services late and when they do get to the facility, the cannot comply with the treatment provided. As one healthcare provider in Taiz explained,

“*When a marginalized woman gets to the center, I give her the needed counseling, write her the medications, and tell her to buy them from the pharmacy. How can she buy them when she has nothing? She won't come back again for therapy. Mothers don't attend the counseling sessions because they don't receive the service nor the medications.”*

#### Distrust and lack of demand

Some services, particularly immunization and family planning, have become less socially and politically acceptable since the conflict escalated. For example, many families are becoming less interested in vaccinating their children as benefits of preventive health services are not as tangible as food assistance and treatment of acute conditions. Reluctance is exacerbated by rumors regarding foreign agendas pushing for vaccination and family planning.

*“Unfortunately, we have recently witnessed a fall back in interpreting the concept of "family planning". People lack to understand this concept. We are back again to the pre-eighties periods when people were fighting what's called ‘birth control’ which contradicts our religious beliefs.”* -INGO Country Office

There are efforts to mitigate this decline in acceptability through Health Cluster advocacy efforts, health education and awareness campaigns, and these barriers pale in comparison to the challenges presented in procurement, management and transport of essential medical supplies and commodities in many areas.

To reach children that may not have been vaccinated, humanitarian partners have implemented a number of measles and polio immunization campaigns - in some cases integrated with efforts to screen all children under five for malnutrition and refer identified cases of moderate or severe malnutrition for care. Although many key informants cited these campaigns as critical strategies to maintain immunization coverage, others noted challenges in obtaining government support or permissions to reach all areas in need, particularly in de-facto government-controlled areas.

## Discussion

The effectiveness of humanitarian public health action depends on knowledge of the context, understanding of priority needs, and correct and timely identification and implementation of the most appropriate interventions [37]. Recent program evaluations in other areas of Yemen have shown that there is continued demand for family planning, maternal and newborn health care and utilization increases when these services are supported by programs and resources [32]. Increasing attention to RMNCAH, particularly reproductive, maternal and newborn health services, requires strategic advocacy within and outside of Yemen. Restoration of services across the continuum of care should be viewed as an essential means of improving humanitarian response. This includes continued support for essential RMNCAH+N services outlined in national and international standards, including investments in preventing unintended pregnancies and addressing the health systems building blocks needed to increase availability and quality of essential maternal, newborn and child health services [38].

Emergency health and nutrition programs, together with other large-scale humanitarian assistance mechanisms, have played a major role in mitigating complete health system collapse, but may present challenges in the long run as they are perceived as solely focusing on maintaining essential services during the emergency period, without consideration for health service quality, sustainability or recovery needs. As donors broaden engagement strategies to include restoration of institutional capacity and expansion of service delivery mechanisms, initial steps could be reviewing the quality of training activities, technical supervision and monitoring, and investing in infrastructure repair, workforce development, supply management and information systems where feasible, or at least supporting situation analyses, forecasting and costing of medium/long term recovery needs. In the meantime, standardization of incentives and support systems for reproductive, maternal, newborn and child healthcare providers is essential to maintaining the skills-mix needed to provide essential services for women and children. Donors should continue to invest in community-based RMNCAH+H service delivery strategies to bring care closer to women and children, and in strengthening linkages between community and facility-based services as the system recovers. UN agencies and international and national NGOs implementing integrated primary care strategies must harmonize incentives for provision of essential healthcare services, ensure renewed attention to reproductive and maternal health needs, as current models are designed with greater emphasis on child health and nutrition concerns. The bulk of these services are provided by female health workers, who are in short supply in many areas and face exacerbated inequities and risks in conflict-affected settings [39, 40]. Mobile clinics and outreach strategies may be suitable for preventive services and outpatient case management of chronic conditions, but are unlikely to be able to deliver a comprehensive package of preventative and curative care, [41] meaning that gaps in availability of care for women during pregnancy, childbirth and the postpartum period will persist until community-based midwifery programs are expanded and access to quality, facility-based services are restored.

Factors affecting the design and implementation of RMNCAH+N service delivery in Yemen are not unique to this crisis [42]. In large-scale complex emergencies, needs invariably exceed resources and insecurity often hinders humanitarian access. Fragile health systems are further weakened, with shortages and inequitable distribution of human resources, and weak or disrupted supply chains and management systems. Economic decline and food insecurity are not uncommon [43, 44]. The conflict, complex political geography, severe food insecurity, devastated economy and severity of the humanitarian crisis, however, compounded these challenges in Yemen, placing enormous strains on an already weak and fragmented health system. The confluence of ongoing large-scale conflict, infectious disease, and food insecurity in a country with endemic poverty and high prevalence of gender-based violence has placed women and children at increased risk of negative health outcomes, including unwanted pregnancy, obstetric complications and related mortalities and morbidities, and preventable maternal and child mortality [45, 46].

Lessons learned from Yemen will be important for guiding future humanitarian responses. First, the cholera epidemic in Yemen had a negative effect on health services addressing issues beyond cholera control and treatment, including RMNCAH+N. While the number of suspected cholera cases were likely significantly overestimated, the epidemic was still massive, and human resources, technical expertise, funds and logistics were strongly prioritized towards the cholera response [26]. There was a lull between cholera outbreaks where increased attention to the sidelined aspects of RMNCAH and its interactions with severe acute malnutrition could have occurred. At the time of writing this article, however, the cholera epidemic has once again dramatically increased. Examining trade-offs in prioritizing nutrition and infectious disease control efforts over other services can guide strategies for addressing routine health needs during epidemics in conflict settings such as cholera in Yemen and Ebola in the Democratic Republic of Congo.

Second, availability, retention and motivation of qualified health workers remains a major challenge. Coordination and harmonization of financial and non-financial incentives for healthcare providers is essential to maintaining the skills-mix needed to provide essential services for women and children.

Efforts to expand the community-based health workforce and ensure staff at functional facilities have skills needed to provide quality care are challenging due to the insecurity and reduced accessibility in many parts of the country, and are not sufficient to meet population needs. For example, community midwives are a critical cadre for maintaining essential health services, and global studies have noted the benefits of community midwifery in increasing access for marginalized and hard-to-reach communities [39]. The size of the current workforce is limited, however, and community midwifery education, which was only operational in select areas at the start of the conflict, is a 36-month course [39, 47]. Continued expansion of community health volunteer networks as well as investments in midwifery education, and medical and nursing diploma programs, as soon as possible will be critical to health system recovery.

Third, investment in strengthening supply chains and management systems is also critical. Although many of the bottlenecks in ensuring availability of essential RMNCAH+N commodities are political, this essential health system building block was poorly functioning before the war. For example, before the escalation of conflict in 2015, all contraceptive supplies in the country were donor-funded and managed separately from the MoPHP supply chain for essential health commodities, making it vulnerable to disruption [48]. Although supply shortages and irregularity were common themes in qualitative interviews and focus group discussions, no participants made reference to any commodity security initiatives inaugurated prior to the escalation of conflict, suggesting that the small progress made in this area is now negligible and similar initiatives are unlikely to yield results in the current political environment.

Finally, more must be done to remove cost barriers to RMNCAH+N care seeking. Foundations and precedent for this are in place. Current emergency health and nutrition programs are using multiple mechanisms to assist with health facility operational costs, including providing in-kind resources (e.g. fuel), providing lump sum payments to cover water, electricity, security and cleaning needs of facilities, and providing either individual or facility-level performance-based incentive payments. Although this may mitigate the need for facilities to charge user fees to cover basic operating costs, it does not address all financial barriers to care seeking. Demand-side financing strategies have been used in other humanitarian settings and may be a promising strategy, if well-coordinated with emergency social protection assistance coordinated by UNICEF and other cash based humanitarian assistance programs [49–51]. Safe motherhood and family planning voucher programs in two governorates were discontinued as conflict escalated, but had promising results and may be worthy of adaptation [52, 53].

This case study has a number of limitations. First, data available for analysis of trends in health service utilization or coverage over time was extremely limited; with both desk review and qualitative data collection yielding strong cautions about the completeness and reliability of routine data sources. The national HMIS is not functional, as is often the case in humanitarian emergencies, and data collected by humanitarian agencies to inform programming is not easily aggregated to facilitate analysis of trends over time. As a result, although we triangulated responses from government authorities, humanitarian agencies and health workers with secondary data sources to minimize risk of political bias, perceived impacts of conflict on health service coverage and quality could not be verified. Second, no private sector facilities were visited, which narrows the scope of our case study despite their expanding and vital role during the crisis. Third, no RMNCAH+N service beneficiaries were interviewed; it is possible that observations and opinions expressed by community health workers, as well as facility-based health care providers and humanitarian agency staff, do not fully reflect the experiences of potential care seekers in the study sites. Fourth, because this study was designed to examine factors affecting RMNCAH+N service delivery across the continuum of care, qualitative interviews did not probe in-depth about specific services. A more focused study would allow for comprehensive examination of factors affecting availability, quality and effectiveness of specific health services during the ongoing conflict. Last, transcription/translation of qualitative findings by non-Yemenis and analysis in English means that some nuances of dialect may be missed. Nevertheless, it is among the first studies to systematically analyze the factors affecting delivery of health services for women and children since the escalation of conflict in 2015 and sheds light on the challenging trade-offs that must be considered in health program planning and implementation in complex humanitarian crises.

## Conclusions

The impact of conflict on health services for women and children in Yemen is profound and multifaceted. Although there has been notable attention to select aspects of maternal, newborn, and child health care, these efforts have been overshadowed by the urgency of cholera epidemic control and widespread severe acute malnutrition. Urgent and concerted action is needed to remove political barriers to address health needs and ensure restoration of a comprehensive package of RMNCAH services through investment in the retention and motivation of skilled health workers, further decentralization of health services, removal of cost barriers to care, and strengthening of supply chains and management systems. Attention to these issues is needed now, with simultaneous investments in peace, security, emergency relief, and development to ensure every woman, newborn, child and adolescent in Yemen is able to realize their rights to health and opportunity. Lessons learned from Yemen as to how to address ongoing primary health care needs during massive epidemics in conflict settings, particularly for women and children, will be important to support other countries in similar situations in the future.

## Supplementary information

**Additional file 1.** Documents and Data Sources Reviewed.

**Additional file 2.** Data Collection Tools.

## Data Availability

Data is available upon reasonable request from the corresponding author.
