# Supplementary material for: Reproductive, maternal, newborn and child health service delivery during conflict in Yemen: a case study
Source: Confl Health. 2020 May 27;14:30. doi: 10.1186/s13031-020-00269-x (PMC7254736; doi:10.1186/s13031-020-00269-x)
Supplement: Supplementary file 1 — Additional file 1. Documents and Data Sources Reviewed. [file 13031_2020_269_MOESM1_ESM.docx]

**Documents and Data Sources Reviewed**

**Conflict data sources**

- ACLED, Jan 2016-Oct 2018
- UCDP
- xSub
- Yemen Data Project, Mar 2015 – Oct 2018

**Health and Population data sources**

*National*

- 2006 Multiple Indicator Cluster Survey Report and Dataset (UNICEF)
- 2012 Comprehensive Food Security Survey Report (WFP)
- 2013 Demographic and Health Survey
- 2014 Comprehensive Food Security Survey Report (WFP)
- 2014 Annual Health Statistics Report (MoPHP)
- 2016 Emergency Food Security and Nutrition Assessment (WFP)
- 2016 Service Availability and Health Facilities Functionality in 16 Governorates Report (WHO)
- 2016 Humanitarian Needs Overview Dataset (OCHA)
- 2016 Population Projection (Central Statistics Office)
- 2017 Population Projection (Central Statistics Office)
- 2018 Population Projection (Central Statistics Office)
- 2018 Heath Resources and Service Availability Mapping Functionality Dashboard (WHO)
- Task Force on Population Movement Reports and Datasets, Rounds 1-17 (IOM)

*Sub-national*

- 2012 Aden Nutrition Survey Report
- 2014 Taiz Smart Survey Report
- 2015 Aden Nutrition and Mortality Survey Report
- 2017 Taiz Emergency WASH and Nutriton Survey Report
- 2018 Aden IYCF Monthly Database
- 2018 Aden MAM Database
- 2018 Aden SAM Database

**SitReps**

- ACAPS briefing notes (April 2015, July 2015, March 2017, May 2017, November 2017, December 2017, March 2018, August 2018)
- OCHA Humanitarian Bulletin 1-30 (2015-2018)
- OCHA Humanitarian Updates 1-30 (2018)
- OCHA Humanitarian Access Snapshots (2017-2018)
- UNFPA Situation Reports (2016-2018)
- UNICEF Situation Reports (2017-2018)

**Coordination Reports**

- OCHA Humanitarian Needs Overview (2015, 2016, 2017, 2018)
- OCHA Humanitarian Response Plan 2016, 2017, 2018)
- Health Cluster Bulletin (2017-2018)
- Nutrition Cluster Bulletin (2016-2018)

**Thematic Reports**

Alterman JB. [Aid and conflict: pitfalls in Yemen](https://csis-prod.s3.amazonaws.com/s3fs-public/publication/180820_Alterman_Yemen_FINAL.pdf?4uoy_B2EGb4E5H5kVQfcc3BY5CRiDVlh). CSIS Brief. August 2018.

El Deek, A., Abdo Ahmed, S., Salem, O., and Hart, K.. [Pathways for Peace and Stability in Taiz, Yemen: An Analysis of Local Conflict Dynamics and Windows of Opportunity](https://www.sfcg.org/wp-content/uploads/2018/07/Yemen_whitepaper_dr2_July-2018.pdf). Search for Common Ground. 2018

Office of the High Commissioner for Human Rights. (2018) Situation of human rights in Yemen, including violations and abuses since September 2014. 39^th^ Session ([A/HRC/39/43](http://undocs.org/A/HRC/39/43.)).

Salisbury P. [Yemen: National Chaos, Local Order](https://www.chathamhouse.org/sites/default/files/publications/research/2017-12-20-yemen-national-chaos-local-order-salisbury2.pdf). Chatham House Research paper. December 2017.

Salisbury P. Y[emen’s Southern Powder Keg](https://www.chathamhouse.org/sites/default/files/publications/research/2018-03-27-yemen-southern-powder-keg-salisbury-final.pdf). Chatham House Research Paper. March 2018.

International Crisis Group. [Report 193: How to Halt Yemen’s Slide into Famine.](https://www.crisisgroup.org/middle-east-north-africa/gulf-and-arabian-peninsula/yemen/193-how-halt-yemens-slide-famine) 21 November 2018.

Sharp JM. Yemen: [Civil War and Regional Intervention](https://fas.org/sgp/crs/mideast/R43960.pdf). Congressional Research Service. 24 August 2018.

**Journal articles**

1. Al-Badani A, Al-Areqi L, Majily A, Al-Sallami, Al-Madhagi, Al-Kamarany MA. Rotavirus diarrhea among children in Taiz, Yemen: Prevalence-Risk Factors. Internatioanl Journal of Pediatrics.2014.
2. Almahbasi T, Aljunid AM, Ismail A. Health labor market requirements of health professional education in Yemen.Eastern Mediterranean Health Journal. 23 (6): 2017.
3. Almahbasi T, Aljunid AM, Ismail A. Health labor market requirements of health professional education in Yemen.Eastern Mediterranean Health Journal. 23 (6): 2017.
4. Al-Mudhwahi A. Role of integrated outreach activities in improving nutritional status among under-five children in Yemen. Journal of Nutritional Science and Vitaminology. 61. S60-62. 2015
5. Al-Seroui A, AL Rabee A, Bin Afif M, Al Rukeimi A. Reducing maternal mortality in Yemen: Challenges and lessons learned from baseline assessment. IJGO. 2009
6. Alssamei FA, Al Sonboli NA, Aljumaim FA, Alsayaad NS, Al-Ahdal MS, Higazi TB, Elagib AA. Assessment of Immunization to Hepatitis B Vaccine among Children Under 5 Years in Rural Areas of Taiz, Yemen.
7. Amad MA, Al-Eryani L, Al Serouri A, Khader Y. Evaluation of outpatient therapeutic program (OTP) for treatment of severe acute malnutrition in Yemen: a focus on treatment default and its risk factors. Journal of Evaluation in Clinical Practice. June 2017
8. Badahdah, A. Attitudes toward restricting the sexual and reproductive rights of women living with HIV infection in Yemen. Journal of the Association of Nurses in AIDS Care. 27, 180-187.2016
9. BaSaleem HB, Baamer A, Al-Sakkaf K, Bin Briek A, Saeed A. Maternal and Neonatal Health Care Knowledge Among Yemeni Community Midwives: A Community-Based Cross-Sectional Study. Research Journal of Obstetrics and Gynecology. 2017
10. Boddam-Whethem L, Xaher G, Al-Kubati E, Garter AC. Vouchers in fragile states: Long-acting reversible contraception in Yemen and Pakistan. Global Health Science and Practice. 4 (2). 2016
11. Briody C, Rubsenstein L, Roberts L, Penny E, Keenan W, Horber J. Review of attacks on health care facilities in six conflicts of the past decades. Conflict and Health. 12:19. 2016
12. Camacho A, et al Cholera epidemic in Yemen, 2016-2018: an analysis of surveillance data. Lancet. 2018
13. De Jong J, Bahubaishi N, Attal B. Effects of reproductive morbidity on women's lives and costs of accessing treatment in Yemen. Reproductive Health Matters. 2012
14. DeSouza L. Correlates of child undernutrition in Yemen. Bandung: Journal of the Global South.4 (3): 2017
15. Dodd R, Huntington D, Hill P. Programme alignment in higher-level planning processes: a four-country case-study for sexual and reproductive health. Int J Health Plann Mgmt. 2009
16. El Bcheraoui C, Jumaan A, Collison M, Daoud F, Mokdad A. Health in Yemen: Losing Ground in War Time. Globalization and Health. 14(42): 2018.
17. Fahmy K, Hampton L, Langar H, Patel M, Mir T, Soloman C, Andreas H, Yusuf N, Teleb N. Introduction of inactivated polio vaccine, withdrawl of type 2 oral polio vaccine, and routine immunization strengthening in the Eastern Mediterranean Region. Journal of Infectious Diseases. 2016
18. Gopalan SS, Das A, Howard N. Maternal and neonatal service usage and determinants in fragile and conflict-affected situations: a systematic review of Asia and the Middle East.Women's Health. 17 (20): 2017.
19. Grainger CG, Gorter AC, Al-Kobati E, Boddam-Whetham L. Providing safe motherhood services to underserved and neglected populations in Yemen: the case for vouchers.Journal of International Humanitarian Assistance. 2(6): 2017.
20. Kempe A, Alwazer FNA, Theorell T. Women's authority during childbirth and Safe Motherhood in Yemen.Sexual and Reproductive Healthcare. 2010.
21. Kempe A, Alwazer FNA, Therell T. The role of demand factors in utilization of professional care during childbirth: perspectives from Yemen.ISRN Obstetrics and Gynecology. 2011.
22. Kempe A, Theorell T, Alwazer FNA, Christensson K, Johansson A. Yemeni women's perception of own authority during childbirth: What does it have to do with achieving the Millenium Development Goals?Midwifery. 29: 2013.
23. Kempe A, Theorell T, Alwazer FNA, Taher SA, Christensson K. Exploring women's fear of childbirth in a high maternal mortality setting on the Arabian Peninsula.Global Mental Health. 2(e10): 2015
24. Kizler R, Hollins Martin CJ. Could introducing vacuum delivery into the education curriculum of community midwives in Yemen improve maternal and neonatal mortality and morbidity outcomes? Nurse Education in Practice. 13. 2003.
25. Obadi MA, Taher R, Qayad M, et al. Risk factors of stillbirth in Yemen. *Journal of neonatal-perinatal medicine* 2018;11(2):131-36.
26. Qirbi N, Ismail S. Health system functionality in a low-income country in the midst of conflict: the case of Yemen. Health Policy and Planning. 32. 2017
27. Raslan R, Sayegh S, Chams S, Chams N, Leone A, Hussein IH. Re-emerging vaccine preventable diseases in war-affected peoples of the Eastern Mediterranean Region - An Update. Frontiers in Public Health. 2017
28. Saleh S, Alameddine MS, Natafgi NM, Maaria A, Sabri B, Nasher J, Zeiton M, Ahmad S, Siddiqi S. The path towards universal health coverage in the Arab uprising countries: Tunisia, Egypt, Libya and Yemen. Lancet. 2014
29. Sharag MF, Rashad AS. Regional inequalities in child malnutrition in Egypt, Jordan and Yemen: a Blinder-Oaxaca decomposition analysis.Health Economics Review. 6(23): 2016.
30. Teleb N, Hajjeh R. Vaccine preventable diseases and immunization during humanitarian emergencies: challenges and lessons learned from the Eastern Medierranean Region. EMHJ. 22(11): 2016.
31. Vevers M, Narra R. Treating cholera in severely malnourished children in the Horn of Africa and Yemen.Lancet. 2017
