# Supplementary material for: Reproductive, maternal, newborn and child health service delivery during conflict in Yemen: a case study
Source: Confl Health. 2020 May 27;14:30. doi: 10.1186/s13031-020-00269-x (PMC7254736; doi:10.1186/s13031-020-00269-x)
Supplement: Supplementary file 2 — Additional file 2. Data Collection Tools. [file 13031_2020_269_MOESM2_ESM.docx]

**Tool A: Interview Guide for Healthcare Providers**

1. **Complete interview details.**

Date (dd/mm/yyyy): |___|___|:|___|___|:|___|___|___|___|

Province/Zone:

- San’aa
- Aden
- Taiz

Start time |___|___|:|___|___| Interview #: ______________

End time |___|___|:|___|___| Audiofile #: _______________

Name of Interviewer: ____________________________________

Name of transcriber: ____________________________________

Date of transcription: |___|___|:|___|___|:|___|___|___|___|

1. **Interview Guide Description:**

This Guide contains four sections to be completed depending on the roles of the participant:

- Section 1: Introduction
- Section 2: Reproductive, maternal, newborn, child and adolescent health services
- Section 3: Current events الأحداث الأخيرة, and Population Movements
- Section 4: Closing interview

These questions will help us learn about how health services for women and children are delivered in in Yemen since the beginning of the conflict in March 2015. Questions do not have any right or wrong answers.

The interview should take no more than 90 minutes.

**Guide for Interviewer**

- This interview guide is not meant to be used verbatim and not all questions will be relevant to all respondents.
- The main questions are in bold. Probes are preceded by bullets.
- Questions are not meant to be asked in the same sequence. It is expected that participants may answer questions that appear later in the questionnaire as part of their response to earlier questions. In case that happens, feel free to probe around the topic as it comes about. However, make sure that the initial question was also answered (if relevant to your respondent).
- The probes included here are only suggestions. You can use other silent and verbal probes as necessary. If something is unclear, use clarifying questions and encourage participants to elaborate through mirror or reflective probes.
- The questions here cover a large array of services. If your interviewee has already clarified that some services are out of their scope, you can skip those questions later (given they do not pertain to topics like coordination and joint programming for example).
- There are hints and comments not meant to be shared with interviewee, but rather as guidance for you. Those comments will be in italic and/or in a box.
- Words between brackets like [governorate/district] or [role] should be replaced by the individuals own role or region
- For questions intended only for directors of health facilities or hospital managers and not for frontline providers, they will be preceded by **[FOR MANAGER AND DIRECTORS].**

**Interview Guide for Facility-Based Healthcare Providers**

Section 1: Introduction

- 1. **Please tell me about your role(s) at this facility.**

*Probes:*

- - Can you describe a typical day in your practice?
  - How long have you been working at this facility? In this position?
  - What are your primary responsibilities?
  - Are you involved in any community outreach activities, or are all of your responsibilities facility-based?
  - What motivates you to do this work?

**1.2 Can you describe your educational background**

Section 2: RMNCAH+N Services

**2.1 We would like to understand the health and nutrition services provided for women and children in this area. Can you please describe what services are available?**

*Service list:*

- Reproductive, Maternal and newborn health services
- Child and adolescent health services
- Nutrition services
- Community-based services
  1. **[FOR MANAGER AND DIRECTORS] For the services mentioned before, who provides them in this facility, and how often they are available?**
  2. **Can you describe any support you receive to improve the availability and quality of health services for women and children at this facility and who supplies it?**

*Probe:*

- Incentives
- Training
- **[FOR MANAGER AND DIRECTORS]** Supplies
- **[FOR MANAGER AND DIRECTORS]** Equipment
  1. **How did the current situation affect your salary/compensation?**
  2. **How do you assess the health and nutrition services for women and children in your facility?**

*Probe:*

- How do you think the services are going?
- How did they change and why?
  1. **Has there been any change in the availability and level of healthcare delivery of facility and community-based services for women and children since the beginning of the ongoing conflict? Please name some of the most significant changes and when they occurred.**

**2.7 [FOR MANAGER AND DIRECTORS] Can you describe how health staffing changes in your facility have affected what health services are provided?**

*Probe:*

- - Does the availability of certain cadres of health workers influence the type of health services you provide or how you provide them? If so, please explain.
  - Is there shortage in staffing that impairs health service provision (this can include doctors, nurses, midwives, etc.? How did it affect your responsibilities and role in this facility?

**2.8 Can you describe how infrastructure, equipment and supply/medicine availability have affected what health services have been provided?**

*Probe:*

- - Since March of 2015, were there any concerns about the availability or shortages of commodities for women’s and children’s health? Can you describe any adjustments that were made to specific programs or interventions as a result of commodity availability?
  - Do infrastructure and supply challenges affect any particular aspects of facility or community-based health services more than others? Please explain.

Section 3: Current events الأحداث الأخيرة, and Population Movements

**3.1 How is the current safety situation وضع الأمانsituation in [LOCATION]?**

*Probe:*

- - Does the safety situation vary in different parts of [GOVERNORATE/DISTRICT]?
  - How has the safety situation changed since you have been working at this facility?

**3.2 Can you elaborate on how the current situation in affected you and your work?**

*Probe:*

- How did the safety situation affect your work as a healthcare provider?
- Has it affected your case load at the facility? Why is this?
- How did the day to day tasks of your role change since the conflict?
- Were you personally or your family affected by the crisis? Please explain.

**3.3 [Since March 2015/SINCE YOU BEGAN THIS POSITION], how have violence, safety situation and population movements affected the provision of health services for women and children?**

*Probe:*

- - How does security affect family’s decisions to seek care and ability to access health facilities? How do security conditions affect your ability to do your job?
  - How does the security situation affect the costs of providing services?
  - How does the security situation affect the availability of medicines and supplies?
  - How does the security situation affect the level and type of support you receive?
  - Have there been any direct threats or acts of violence against this facility or its employees?
  - Are there other ways that the security situation affects the availability and quality of facility or community-based health services for women and children?
  1. **Have there been any other events or issues that have affected the services provided to women and children at this facility?**

*Probe:*

- - Has this area had a large number of people forcibly displaced or influx of families displaced from other areas? If so, can you describe how this affected reproductive, maternal, newborn, child and adolescent health service provision at this facility?
  - Have there been any disease outbreaks or epidemics since you have been working in this position? If so, can you describe how this affected reproductive, maternal, newborn, child and adolescent health service provision at this facility?
  - Has physical infrastructure (roads, health centers, referral facilities) been affected by the conflict? If so, under what circumstances?
  - Have lines of communication, referral, and/or supply chains been disrupted by conflict at this facility?
  - Does your facility provide any community outreach services, and if so, are they still functioning?

**3.5 How do you and your colleagues cope with these challenges?**

*Probe:*

- - Are there any innovative approaches you have used to try to overcome these obstacles? How successful have they been?

Topic 4: Challenges and Opportunities

**4.1 In your opinion, how well do the services provided at this facility meet the needs of women and children in this area?**

*Probe:*

- - If you or your children needed care, would you be want them to receive it here? If no, why? And where would you take them?
  - Are there any subpopulations (depending on their age, gender, ethnicity, religious sect, etc.) that are particularly hard to reach with services?
  - Can you describe any special efforts to access these populations?

**4.2 what would make service delivery in your facility better?**

Section 5: Closing

- 1. **Before we end, do you have any additional thoughts on service delivery strategies and challenges for women and children that were not shared?**

That is the end of our interview today. Thank you so much for your time. Do you have any questions for me? *[Answer any questions.]*

**Tool B: Focus Group Discussion Guide for Community Midwives & Community Health Volunteers**

1. **Complete interview details.**

Date (dd/mm/yyyy): |___|___|:|___|___|:|___|___|___|___|

Governorate:

- Sana’a
- Aden
- Taiz

Start time |___|___|:|___|___| Interview #: ______________

End time |___|___|:|___|___| Audio file #: _______________

Name of Interviewer: ____________________________________

Name of transcriber: ____________________________________

Date of transcription: |___|___|:|___|___|:|___|___|___|___|

1. **Interview Guide Description:**

This Guide contains five sections to be completed depending on the roles of the participant(s):

- Topic 1: Introduction, organizational expertise, and scope of work
- Topic 2: Reproductive, maternal, newborn, child and adolescent health services
- Topic 3: Current events الأحداث الأخيرة, and Population Movements
- Topic 4: Challenges and Opportunities
- Topic 5: Closing interview

These questions will help us learn about how health services for women and children are delivered in conflict-affected areas of eastern DRC. Questions do not have any right or wrong answers.

The discussion should take no more than 90 minutes.

**Discussion Guide for Community-Based Healthcare Providers**

Welcome to our group discussion. My name is ______________ and I will be moderating today’s session. Joining me today are ­­­­­­­­­­___________ and ____________, who will be taking notes and will be here to assist me. This is an open discussion, and we want each person to participate. The goal is to have everyone contribute to the discussion. We encourage you to express your thoughts and opinions freely. I might interrupt at points during the discussion to make sure we have enough time to cover all topics. If you don’t understand a question, please let us know. We are here to ask questions, listen, and make sure everyone has a chance to share. I would like to use a recording device to record our discussion because the note taker will not be able to write down all that you say. We also ask that everyone speak up so that the recording can pick up your voice. You will not be identified by name in any of the notes from this session.

Here are a few ground rules before we start:

- First, please turn all cell phones to silent.
- Please speak clearly so that our tape recorder can pick up your voice.
- Please speak one at a time.
- Please do not have side conversations
- Please give each other a chance to speak
- There are no right or wrong answers, and we will have different points of view. We encourage you to talk to each other, to add thoughts to others’ comments, and to share reactions or disagreements with respect.
- And we ask that you please respect the privacy of everyone here and do not share the content of this discussion outside this room.

Topic 1: Introduction

- 1. **To start our discussion, can you describe the role of a skilled birth attendant and how you became involved in this work?**

*Probe:*

- What training did you receive to become a community health worker?
- What technical support or supervision do you receive (from nearby facilities, the Ministry of Health, NGOs)?
- What is a typical day of work for a community health worker?
- How did your roles change since the beginning of the current conflict in March 2015?
  1. **What makes you most proud to be a community health worker?**

Topic 2: RMNCAH+N Services

- 1. **We would like to understand more about the health and nutrition services available for women and children in your communities. Can you please describe the services available at the facility or community level?**

*Probe:*

- - Reproductive and maternal health services
  - Newborn health services
  - Child health services
  - Adolescent health services
  - Nutrition services
  - Other services
  1. **We are interested in learning how the availability and quality of services has changed since March of 2015. Have you observed any noteworthy changes in your communities? Please explain.**

Topic 3: War, Safety and Population Movements

**We understand that conflict and insecurity can present many challenges for health workers in [Governorate].**

- 1. **How do you feel about your safety in [Governorate] currently?**
  2. **How has the security situation changed since the beginning of the conflict in 2015?**
  3. **How does the changing security situation affect your job as a community health worker?**

*Probe:*

- Are you still reaching the same families? Why?
- Did this affect how you charge for your services
- How are the support you receive from UN, NGOs, government?
  1. **How does it affect when and where families in your communities decide to seek care?**
  2. **Have there been any other events or issues that have affected the way health services are provided to women and children in your communities?**

*Probe:*

- - Has this area had a large number of people forcibly displaced or influx of families displaced from other areas? If so, can you describe how this affected reproductive, maternal, newborn, child and adolescent health service provision at this facility?
  - Have there been any communicable diseases such as diphtheria or cholera or meningitis or epidemics since you have been working in this position? If so, can you describe how this affected health service provision?
  - Other things affecting services?

Topic 4: Challenges and Opportunities

- 1. **In your opinion, how well do services currently provided in [Governorate] meet the needs of women and children in this area?**

*Probe:*

- - What do you think about services in the facilities?
  - How about services in the community?
  - Are there any subpopulations that are particularly hard to reach with services?
  - Can you describe any special efforts to access these populations?
  1. **What are the organizations supporting community workers?**

*Probe:*

Are there differences in the guidelines or tasks requested from those organizations?

- 1. **As a community health worker, what are the greatest challenges you face in providing quality health services to women and children in your communities?**
  2. **What kind of additional training or resources have been made available since the beginning of the conflict?**

Topic 5: Closing

**5.1 Before we end, does anyone have any additional thoughts on what could be done to improve services for women and children?**

Thank you again for your help and for sharing your opinions with us today. We really appreciate your time and contribution. Feel free to ask any questions.

**Tool C: Interview Guide for Health Officials**

1. **Complete interview details.**

Date (dd/mm/yyyy): |___|___|:|___|___|:|___|___|___|___|

Governorate:

- Sana’a
- Aden
- Taiz

Start time |___|___|:|___|___| Interview #: ______________

End time |___|___|:|___|___| Audio file #: _______________

Name of Interviewer: ____________________________________

Name of transcriber: ____________________________________

Date of transcription: |___|___|:|___|___|:|___|___|___|___|

1. **Interview Guide Description:**

This Guide contains four sections to be completed depending on the roles of the participant:

- Section 1: Introduction
- Section 2: Reproductive, maternal, newborn, child and adolescent health services

(including planning/prioritization, health workforce, and supply chain management)

- Section 3: Effects of conflict/war
- Section 4: Other topics
- Section 5: Closing interview

These questions will help us learn about how health services for women and children are delivered in in Yemen since the beginning of the conflict in March 2015. Questions do not have any right or wrong answers.

The interview should take no more than 90 minutes.

**Guide for Interviewer**

- This interview guide is not meant to be used verbatim and not all questions will be relevant to all respondents.
- The main questions are in bold. Probes are preceded by bullets.
- Questions are not meant to be asked in the same sequence. It is expected that participants may answer questions that appear later in the questionnaire as part of their response to earlier questions. In case that happens, feel free to probe around the topic as it comes about. However, make sure that the initial question was also answered (if relevant to your respondent).
- The probes included here are only suggestions. You can use other silent and verbal probes as necessary. If something is unclear, use clarifying questions and encourage participants to elaborate through mirror or reflective probes.
- The questions here cover a large array of services. If your interviewee has already clarified that some services are out of their scope, you can skip those questions later (given they do not pertain to topics like coordination and joint programming for example).
- There are hints and comments not meant to be shared with interviewee, but rather as guidance for you. Those comments will be in italic and/or in a box.
- Words between brackets like [Governorate] or [role] should be replaced by the individuals own role or region. For officials at the national level, ask about Yemen broadly.

**Interview Guide for Ministry of Health Officials**

Topic 1: Introduction

- 1. **Please tell me a bit about yourself and your professional background**
  2. **Please tell me about your role as [POSITION] with [DEPARTMENT].**

*Probe:*

- - How long have you been working in this position?
  - Can you describe the geographic areas you are responsible for in your position?
  - Can you describe your role in relation to technical and financial decision-making about health services for women and children?

Topic 2: RMNCAH+N Services

**2.1 We are interested in understanding the range of health and nutrition services provided to women and children since March of 2015. Could you tell me which aspects of health services for women and children are within your responsibility as [role].**

*Share the list of areas with interviewee and mark services delivered within their areas of work e.g. reproductive health DG only ask about the first section of the list (you will be using their answers here to ask relevant questions below).*

*Service list:*

| Area | Services | Mark services provided |
| --- | --- | --- |
| Reproductive, maternal and newborn health services | 1. Family planning  2. STI screening and treatment  3. Antenatal care  4. Intrapartum care (normal deliveries)  5. Obstetric complications, including post-abortion care  5. Neonatal care  7. Support for early initiation and exclusive breastfeeding  8. Postnatal care |  |
| Child and adolescent health services | 1. Immunization services 2. Integrated management of child illnesses (IMCI) 3. Other sick child care 4. Adolescent reproductive health services 5. Other adolescent health care services |  |
| Nutrition services | 1. Infant and young child feeding support 2. Growth monitoring 3. Care for moderate acute malnutrition 4. Care for chronic acute malnutrition |  |
| Other health services for women and children |  |  |

- 1. **Has there been any change in services for women and children since the beginning of the current situation الأحداث الحالية منذ الحرب? Please describe some of the most significant changes and when they occurred.**

*Probe:*

- How has availability of services changed?
- Can you describe how access of beneficiaries to services changed?
- How has quality of services changed?
- How has the planning and coordination of services changed?
- Did the same changes occur in all parts of [the country/governorate]?
- Are there certain populations (depending on their age groups, geography, etc) most affected by these changes?
- Have any health needs been created or particularly intensified by the conflict (versus pre-existing chronic needs)?
- Can you describe changes in specific clinical/public health services or how they are provided?
  1. **How do the current services in this [GOVERNORATE/YEMEN] compare to what was planned? Please explain.**

*Probe:*

- Can you share any examples of health and nutrition services at that have been a particular challenge to implement in [GOVERNORATE/YEMEN]?
- Why are these services a particular challenge?
  1. **Over the last year, have you been involved decisions about how and where specific health services for women and children should be provided? If so, what informed and/or continues to inform your decisions on which interventions to deliver?**

*Probe:*

- What information (assessments, data, statistics, reports) is used to determine how facility and community-based health services for women and children should be provided?
- How do global guidelines and scientific literature influence the health services and programs implemented for women and children in [GOVERNORATE/YEMEN]?
  1. **Can you describe how funding availability has affected what health services are provided?**

*Probe:*

- How are health services for women and children in [GOVERNORATE/YEMEN] funded (e.g. government, donor agency, NGO, etc.)?
- Does the source of funding influence the type of services prioritized? If so, please explain.
- Over the last five years, were any services deprioritized or stopped because of financial constraints? If so, please explain.
- Do funding challenges affect any particular aspects of facility or community-based health services more than others?
  1. **How do human resource issues affect health services for women and children in [YEMEN/GOVERNORATE]?**

*Probe:*

- Does the availability of certain cadres of health workers influence the type of health services prioritized? If so, please explain.
- How do health worker recruitment, training and retention strategies influence the type of health services prioritized?
- Were there any reproductive, maternal/newborn, child or adolescent health programs/interventions you hoped to prioritize but couldn’t because of workforce shortages or capacity? Please explain.
- Have there been any changes in job descriptions of clinical cadres, community health workers and/or traditional birth attendants since March 2015?
  1. **How do infrastructure, equipment and supply/medicine availability affect health services for women and children?**

*Probe:*

- Since March of 2015, were there any concerns about the availability or shortages of commodities for women’s and children’s health? Can you describe any adjustments that were made to specific programs or interventions as a result of commodity availability?
- Do infrastructure and supply challenges affect any particular aspects of facility or community-based health services more than others? Please explain.
- Has physical infrastructure (roads, health centers, referral facilities) been affected by the conflict? If so, under what circumstances?
- Have lines of communication, referral, and/or supply chains been disrupted by conflict at this facility?
- How easy is it to deliver supplies to the facilities in [Yemen/your governorate]?
  1. **We know that there are many stakeholders involved in implementation of health services for women and children in [YEMEN/GOVERNORATE]. Can you explain how [GOVERNMENT DEPARTMENT] coordinates health service delivery with UN agencies and NGOs?**

*Probe:*

1. How are different stakeholders (e.g. community leaders, health workers, NGOs, UN agencies, government departments, religious leaders, others) consulted in the design of health strategies, protocols and programs for women and children?
2. In your capacity as [POSITION], have you participated in any humanitarian cluster or coordination meetings? Within which clusters?
3. If yes, what types of decisions are made at these meetings? Can you give an example?
4. Can you describe if those decisions were implemented? If yes, please explain their impact. If not, please describe the challenges to implementation
5. Are there any specific requirements that UN or NGO agencies must adhere to if they want to work within [YEMEN/GOVERNORATE]? Please explain
6. How did the coordination change between before the crisis and after March of 2015? How did this affect planning and implementation of health services for women and children?

Topic 3: Safety-related changes

**3.1 Can you elaborate on how the current safety situation (وضع الأمان) affected you and your work?**

*Probe:*

- - Do safety conditions vary in different parts of [Yemen/GOVERNORATE]?
  - How did the day to day tasks of your role change due the safety concerns?
  1. **Over the last three years, how did the current safety situation (وضع الأمان) affect health service providers?**

*Probe:*

- **[FOR NATIONAL OFFICIALS]:** Have any health workers/facilities or humanitarian agencies been victims of violence or violent threats? Please explain
- How has the conflict affected staff recruitment and retention?
- How do safety issues affect providers’ schedules?
  1. **Over this time, how did the current problems and population movements affect facility and community-based health services for women and children?**

*Probe:*

- How does safety affect availability and quality of services provided?
- How does safety affect family’s decisions to seek care and ability to access health facilities?
- How does safety affect the costs of accessing services?
- Are there other ways that the safety situation affects the availability and quality of facility or community-based health services for women and children?
- If you needed care for yourself or your children, what would you do? Would you consider using government services?
  1. **Are there any populations that are particularly hard to reach? Have there been any efforts to extend services to these populations?**

*Probe:*

- Have there been any large influxes of displaced populations? How has this affected service provision in this [GOVERNORATE/YEMEN]?
- Did you receive any additional support to provide services to these IDPs?
  1. **To what extent are IDPs a factor in deciding how and where health services for women and children are provided?**

*Probe:*

- Are there any populations that are particularly hard to reach? Have there been any efforts to extend services to these populations?
- Have there been any large influxes of displaced populations? How has this affected service provision in this [GOVERNORATE/YEMEN]? Did you receive any additional support to provide services to these IDPs النازحين?

Topic 4: Other questions

- 1. **Are there other major events or issues that have affected the coverage and quality of health services for women and children in [YEMEN/GOVERNORATE/DISTRICT]?**

*Probe:*

- Have disease outbreaks/epidemics affected services for women and children are provided? How?
  1. **What coping mechanisms are you using to maintain services for women and children amid the current problems?**

*Probe:*

- Are any particularly effective? Do they have any recommendations for interventions that would be particularly impactful to mitigate the effects of conflict on service provision?

Topic 5: Closing

1. **Before we end, is there anything else you would like to share about the design and implementation of health services for women and children in [Yemen / GOVERNORATE]?**

That is the end of our interview today. Thank you so much for your time. Do you have any questions for me? *[Answer any questions.]*

**Tool D: Interview Guide for UN and NGO senior management and technical leads**

1. **Complete interview details.**

Date (dd/mm/yyyy): |___|___|:|___|___|:|___|___|___|___|

Province/Zone:

- Sana’a
- Aden
- Taiz

Start time |___|___|:|___|___| Interview #: ______________

End time |___|___|:|___|___| Audiofile #: _______________

Name of Interviewer: ____________________________________

Name of transcriber: ____________________________________

Date of transcription: |___|___|:|___|___|:|___|___|___|___|

Start time |___|___|:|___|___| Interview #: ______________

End time |___|___|:|___|___| Audio file #: _______________

Name of Interviewer: ____________________________________

Name of transcriber: ____________________________________

Date of transcription: |___|___|:|___|___|:|___|___|___|___|

1. **Interview Guide Description:**

This Guide contains four sections to be completed depending on the roles of the participant:

- Section 1: Introduction
- Section 2: Reproductive, maternal, newborn, child and adolescent health services

(including planning/prioritization, health workforce, and supply chain management)

- Section 3: Insecurity and population movements
- Section 4: Challenges and opportunities
- Section 5: Closing interview

These questions will help us learn about how health services for women and children are delivered in in Yemen since the beginning of the conflict in March 2015. We are interested in your experience and perspective here. Questions do not have any right or wrong answers.

The interview should take no more than 90 minutes.

**Guide for interviewer**

- - This interview guide is not meant to be used verbatim and not all questions will be relevant to all respondents.
  - The main questions are in bold. Probes are preceded by bullets.
  - Questions are not meant to be asked in the same sequence. It is expected that participants may answer questions that appear later in the questionnaire as part of their response to earlier questions. In case that happens, feel free to probe around the topic as it comes about. However, make sure that the initial question was also answered (if relevant to your respondent).
  - If carrying out a group interview, the interview might take a longer time. In this case try to focus probing on the participant within the group with the most expertise on the topic.
  - The probes included here are only suggestions. You can use other silent and verbal probes as necessary. If something is unclear, use clarifying questions and encourage participants to elaborate through mirror or reflective probes.
  - The questions here cover a large array of services. If your interviewee has already clarified that some services are out of their scope, you can skip those questions later (given they do not pertain to topics like coordination and joint programming for example).
  - There are hints and comments not meant to be shared with interviewee, but rather as guidance for you. Those comments will be in italic and/or in a box.
  - Words between brackets like [governorate] or [role] should be replaced by the individuals own role or region

**Interview Guide for UN and NGO senior management and technical leads**

Section 1: Introduction, Organizational Expertise and Scope of Work

- 1. **Please tell me about your role(s) with [UN ORGANIZTION/NGO].**

*Probe:*

- - How long have you been working in [GOVERNORATE]?
  - How long have you been working in this position?
  - What are your roles and responsibilities?

*If generalist:* Can you describe any specific responsibilities health or nutrition programs/services for women and children?

- How can you explain interaction with your agencies’ other offices within Yemen?
- What kind of interaction with the agencies’ regional and headquarters office if any? *(if relevant)*
  - What is most rewarding about your job?
  - What is most challenging?
  1. **Does [ORGANIZATION] have expertise in a particular area or type of health service provision? If so, how does that influence your scope of work in YEMEN?**

Section 2: RMNCAH+N services

- 1. **In general, how do you assess the health and nutrition services for women and children in [YEMEN/GOVERNORATE]?**

*Probe:*

- Reproductive health services
- Maternal and newborn health services
- Child health services
- Adolescent health services
- Nutrition services
- Community-based services
- Other services
  1. **What are the strengths and weaknesses of facility and community-based services for women and children?**
  2. **How does [ORGANIZATION] make decisions about how and where specific health services for women and children should be provided?**

*Probe:*

- Can you describe how needs assessments, surveys or routine program data are used in program planning?
- How do global guidelines and scientific literature influence the health services and programs implemented?
- How do current [NATIONAL/LOCAL] policies influence decision making and programming?
- How does the cost or cost-effectiveness of interventions influence decisions on what services and programs are prioritized?
- How are different stakeholders (e.g. community leaders, health workers, other NGO/ UN agencies, government departments, religious leaders, others) consulted in the design of health programs for women and children?
- How do local beliefs and practices influence the way health services and programs implemented for women and children in [YEMEN/GOVERNORATE]?
- How does your mandate affect your ability to support interventions?
  1. **What tradeoffs do you have to consider in deciding how and where specific health services should be provided?**

*Probe:*

- How does the level and source of funding received by your organization influence the type of services and service delivery approaches implemented? Please explain.
- How does the availability of certain cadres of health workers influence the types of services and service delivery approaches implemented? Were there any reproductive, maternal/newborn, child or adolescent health interventions you hoped to prioritize but couldn’t because of workforce shortages or capacity?
- How do the available infrastructure and supply chain mechanisms influence the types of services and service delivery approaches implemented? Can you describe any adjustments that were made to specific programs or interventions as a result of commodity availability?
- How do the presence or activities of other humanitarian actors influence the type of health services and service delivery approaches implemented?
- How was the funding allocated for predetermined health services affected?
- What are other factors or tradeoffs that affect health and nutrition planning and resource allocation for women and children?
  1. **There are many stakeholders involved in implementation of health services for women and children in [YEMEN/GOVERNORATE]. Can you explain how [ORGANIZATION] coordinates planning and implementation of health services with other actors?**

*Probe:*

- In your capacity as [POSITION], have you participated in any humanitarian cluster or coordination meetings?
   If so, Within which clusters?
- If yes, what types of decisions are made at these meetings? Can you give an example?
- Can you comment on the level of implementation of the decisions made?
- Have there been efforts of coordination across clusters relating to RMNCAH+N services?
- Can you describe how [ORGANIZATION] coordinates planning and implementation with government authorities?
- Are there any specific requirements that UN or NGO agencies must adhere to or permissions needed to work within [YEMEN/GOVERNORATE/ZONE]? Please explain.
- Are there any other groups or individuals you must coordinate or negotiate with in order to effectively implement health programs for women and children in [GOVERNORATE/ZONE]?
- Are there examples of joint programming between [organization] and other UN organizations or NGOs?

Section 3: Conflict, Insecurity and Population Movements

**We understand that conflict, insecurity and population movements can make planning and implementing health services in Yemen quite difficult.**

- 1. How does the changing security situation affect [ORGANIZATION] and its staff? Please explain.

*Probe****:***

- How does [ORGANIZATION] manage security challenges?
  1. **[SINCE MARCH 2015/SINCE YOU BEGAN THIS POSITION], how has the current situation affected he provision of health services for women and children?**

*Probe:*

- How does the security situation affect the costs of providing services?
- How does the security situation affect health facility staffing and service availability?
- How does the security situation affect program planning and resource allocation?
- How does the security situation affect program implementation and monitoring?
- How does the security situation affect community-care-seeking?
- Are there other ways that the security situation affects the availability and quality of facility or community-based health services for women and children?
- Did the security situation make your organization change the location, stop or temporary hold up activities in [Yemen/this governorate]?
  1. **How has [ORGANIZATION] adapted programming in response to large movements or influxes of displaced population in [YEMEN/GOVERNORATE/DISTRICT]?**

*Probe:*

- How do population movements (departure/influx/return of IDPs) influence how and where health services for women and children are provided?
- Have you received additional resources or support to provide services to newly displaced populations?
  1. **Are there other major events or issues that have affected the coverage and quality of health services for women and children in [YEMEN/GOVERNORATE/DISTRICT]?**

*Probe:*

- Were there any disease outbreaks/epidemics while you were working here? Can you describe how this affected health and nutrition service provision?

Section 4: Challenges and Opportunities

- 1. **From your perspective, how does your [ORGANIZATION]’s programming in [YEMEN/GOVERNORATE] change since March 2015 compare to what was planned? Please explain.**

*Probe:*

- Why are these services a particular challenge?
  1. **Are there any populations that are particularly hard to reach (based on their age, rural or remote location, ethnicity, sect. etc.)? Have there been any efforts to extend services to these populations?**
  2. **Can you share any examples of specific reproductive, maternal/newborn, child or adolescent health and nutrition services that have been a particular challenge to implement in [YEMEN/GOVERNORATE]?**
  3. **Are there any innovative strategies [ORGANIZATION] has used to ensure availability of quality health services across areas of [YEMEN/GOVERNORATE]?**

*Probe:*

- Facility-based health services for women and children
- Community-based health services for women and children
- Coordination

Section 5: Closing

- 1. **is there anything else you would like to share about the design and implementation of health services for women and children in [YEMEN/GOVERNORATE]?**
  2. **Before we end, we value your contribution to this study today and we are also doing a desk review of health services for women and children in Yemen. DO you have reports or data (including governorate annual health reports, data sets, assessments, etc.) that would be helpful to this effort?**

If so, can I take it now or can you tell me who to follow up with for these documents?

That is the end of our interview today. Thank you so much for your time. Do you have any questions for me? *[Answer any questions]*
